# The Important Role of Endoscopy in Management of Pediatric Pseudomembranous Necrotizing Tracheitis

**DOI:** 10.3389/fped.2020.00360

**Published:** 2020-07-09

**Authors:** Xiling Wu, Lei Wu, Zhimin Chen

**Affiliations:** Department of Pulmonology, The Children's Hospital, Zhejiang University School of Medicine, National Clinical Research Center for Child Health, Hangzhou, China

**Keywords:** necrotizing tracheitis, endoscopy, airway obstruction, mechanical debridement, pediatric

## Abstract

Pseudomembranous necrotizing tracheitis is a rare, but life-threatening cause of central airway obstruction. Here, we reported three cases of pediatric pseudomembranous necrotizing tracheitis. The infectious etiologies were *Staphylococcus aureus* secondary to influenza A virus *and Aspergillus fumigatus*. Endoscopy was used in diagnosis and management of all patients and two patients survived. The improvement in mortality rate of these diseases need early recognition and prompt treatment with mechanical debridement by endoscope and early initiation of broad spectrum antibiotics. Endoscopy is a promising tool to diagnose and remove the pseudomembrane, therefore relieving central airway obstruction.

## Introduction

Pseudomembranous necrotizing tracheitis is a rare, but life-threatening cause of central airway obstruction. Common infectious etiologies included bacterial, fungal, and/or viral infection(s) (1). The risk fact was bacterial infection secondary to influenza virus and long-term use of hormones or antibiotics. Patients usually have hoarseness, cough, dyspnea, wheezing and inspiratory stridor. Diagnosis requires a comprehensive endoscopic examination and biopsy (2). Endoscopy (flexible or rigid) has a very important role in both diagnosis and management of pseudomembranous necrotizing tracheitis. Once patients exhibit signs and symptoms of airway obstruction, mechanical debridement by endoscopy is necessary (2). A delay in treatment can significantly worsen the patient's prognosis. Here, we report three cases of pediatric pseudomembranous necrotizing tracheitis. Endoscopy was used to remove the pseudomembrane in all patients and two patients survived.

## Case Presentation

### Case 1

**Figure 1:**
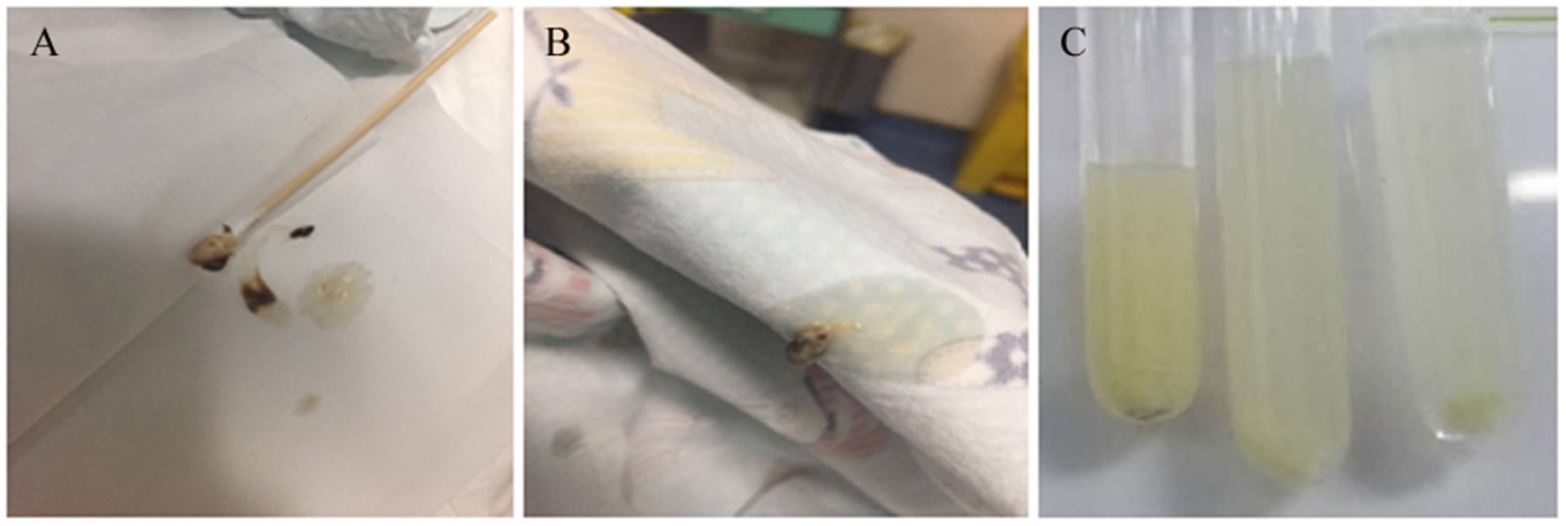
Images of airway secretions or mucous exudates for cases 1 and 2. **(A,B)** Case 1, **(C)** Case 2.

**Figure 2:**
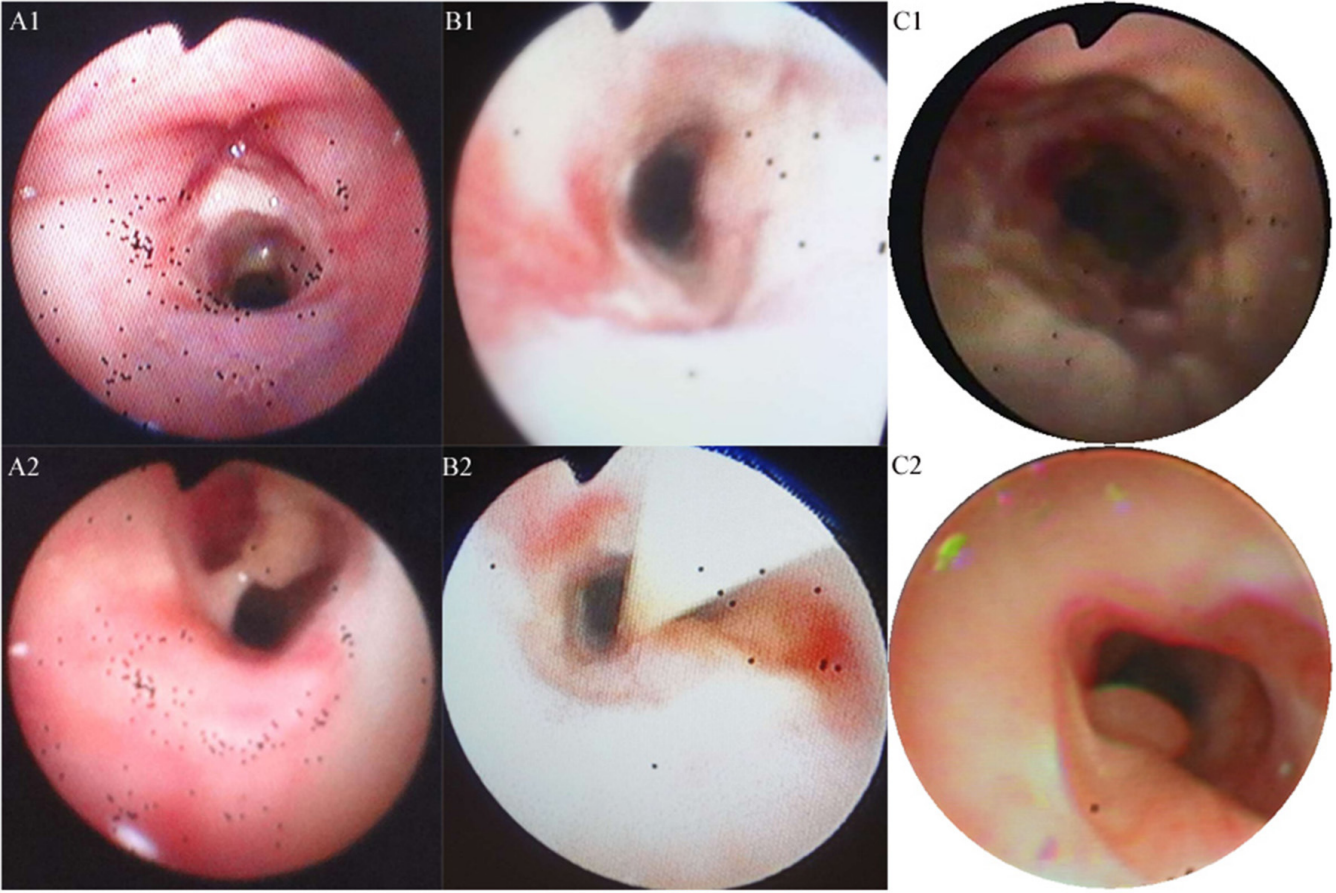
Trancheal bronchoscopy images for the three cases. **(A,B)** Cases 1 **(A1,A2)** and 2 **(B1,B2)** showed substantial purulent secretion and necrotic mucosa. **(C)** Case 3 showed necrotic mucosa during acute phase **(C1)** and irregularly shaped trachea and granulation during the recovery phase **(C2)**.

**Figure 3:**
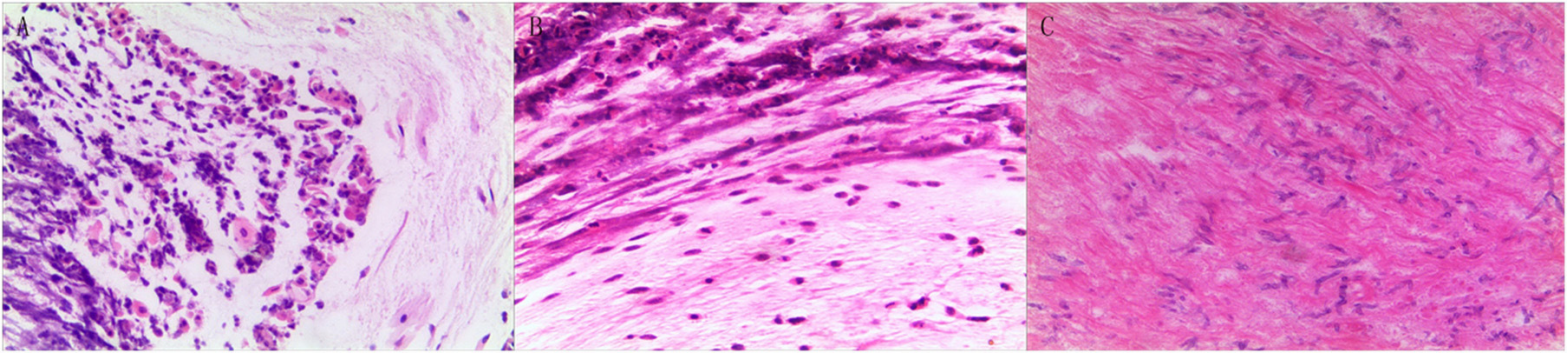
Pathologic results of tracheal biopsy for the three cases. **(A)** Case 1 exhibited neutrophil infiltration and fibrinoid necrosis. **(B)** Case 2, it showed fibrinoid necrosis and purulent cells. **(C)** Case 3 demonstrated fibrinoid exudation and substantial *Aspergillus* load. (Hematoxylin and eosin staining, 200 × magnification).

**Figure 4:**
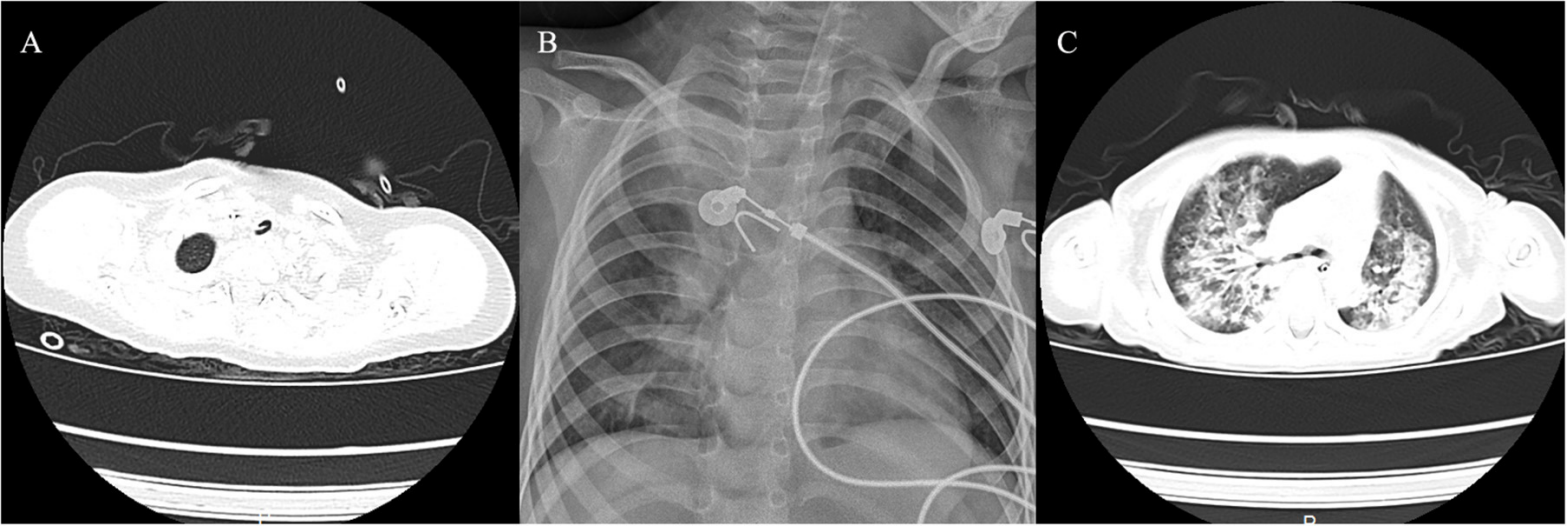
Imaging findings of the three cases. **(A)** Case 1 showed membranous material that was strongly adherent to the tracheal wall. **(B)** Case 2 showed inflammation in bilateral lungs. **(C)** Case 3 had local stenosis of right main bronchus.

A 20-month-old girl presented at our hospital with a history of cough and fever for 2 days, and shortness of breath for 1 day. She also had hoarseness. On admission she was short of breath and exhibited laryngeal stridor. Physical examination revealed that there was no wheezing in both lungs. Computed tomography (CT) demonstrated inflammation in the left lower lobe. Her initial laboratory tests results were as follow: hemoglobin 11.6 g/dL, WBC14.11 × 10^9^ /L, platelet count of 272 × 10^9^ /L, and C-reactive protein (CRP) of 63 mg/L. On the first night of admission, laryngoscope was performed and revealed mucous edema of the larynx. Her situation deteriorated quickly, subsequently requiring intubation. However, her oxygen saturation was still unstable and the airway pressure was high during mechanical ventilation. She received tracheal tube replacement, during which brown solid matter measuring ~1 cm was drawn from her trachea (Figures 1A,B). After tube replacement, her airway pressure decreased and her situation improved after 2 days of meropenem and methylprednisolone treatment Mechanical ventilation was changed to a face mask. Both WBC count and CRP level subsequently decreased to within normal ranges. Sputum bacterial cultures showed a small amount of methicillin-sensitive *Staphylococcus aureus* (MSSA) grow. Sputum antigen testing of influenza (A and B), parainfluenza (I, II, and III), respiratory syncytial virus, adenovirus was negative, and the tuberculin skin test was non-reactive. On day 4, fiberoptic bronchoscopdy showed substantial white matter attached to the anterior commissure of the larynx and trachea (Figures 2A1,A2). Biopsy was performed for pathologic evaluation. Histologic evaluation of biopsy samples revealed flaky necrotic tissue and massive neutrophil infiltration (Figure 3A). On day 5, CT showed a slight high-density cord-like image ~2 cm below the vocal cord and pneumonia in the left lower lobe (Figure 4A). Pathogen detection including fungi, viruses, and bacteria was negative.

The patient had cyanosis and shortness of breath after a gust of coughing on the evening of day 6 after admission. Oxygen saturation decreased to 69%. She had convuls after 2 min and her heart rate decreased with oxygen saturation of 60–70%. We performed tracheal intubation and cardiopulmonary resuscitation. X-ray showed pneumothorax in bilateral lungs and the patient died within 3 h.

To further clarify the causative pathogen, DNA from necrotic tissue was extracted using the DNA Extraction Kit (Omega Bio-Tech Co., Ltd) and determined to be positive for *S. aureus* by the *Staphylococcus aureus* Real Time PCR Kit (Shanghai ZJ Bio-Tech Co., Ltd.).

### Case 2

A 28-month-old girl was admitted with a history of fever and cough for 4 days and progressive shortness of breath for 3 days. Her sputum was brown. Upon admission, she was short of breath and irritable with positive tri-retraction sign. Bilateral respiratory sounds were decreased with rhonchus. Routine blood routine examination revealed WBC of 11.71 × 10^9^/L and CRP of 93 mg/L. X-ray showed inflammation in bilateral lungs (Figure 4B). The patient was hoarseness, shortness of breath, restlessness and the blood gas indicated the decrease of oxygen saturation. She was diagnosed with acute laryngitis with three degrees of laryngeal obstruction, type I respiratory failure, and pneumonia. On the first day of admission, laryngoscopy was performed, which revealed substantial yellowish-white pus in the trachea. Respiratory failure rapidly progressed, subsequently requiring intubation. Bronchoscopy was performed and revealed substantial yellowish-white pus attached to the trachea and right main bronchus (Figures 2B1,B2). Biopsy was then performed (Figure 1C). Alveolar lavage fluid tests indicated the following: lymphocytes 8%, neutrophils 30%, macrophages 63%, and eosinophils <1%. Histologic evaluation revealed fibrin necrotic exudates (Figure 3B). Sputum analysis for viral antigens revealed influenza A positivity. Lavage fluid culture was positive for *S. aureus*. Pathogen detection for fungus or other bacteria was negative. The patient was treated with meropenem, vancomycin, and methylprednisolone. Oseltamivir was also given. Bronchoscopy was performed four times to remove the pseudomembrane and her condition improved. However, on day 8 of admission, her oxygen saturation decreased and she was suspected of having mucous membrane exfoliation. A white sputum bolt sample measuring 2.5 cm was removed by rigid microscopy and the patient's oxygen saturation increased to within normal ranges.

On day 10, the patient's tracheal tube was removed and bronchoscopy was performed seven consecutive times. The tenth bronchoscopic examination showed clear reduction in endotracheal secretion and the mucous membrane was a normal color. On day 32, she was discharged from our hospital.

### Case 3

A 19-month-old girl presented at our hospital with a history of recurrent fever and cough for 2 weeks. She was diagnosed with pneumia by a local hospital and had received azithromycin, ceftriaxone, and methylprednisolone treatment. However, her fever and cough persisted. Upon admission at our hospital, her respiratory rate was 70 breaths/min and body temperature was 39.7^o^C. Her eyelids were swollen and she had a needle-like rash over her entire body. Chest examination revealed crackles in two lung fields. Laboratory examinations revealed WBC count of 2.5 × 10^9^/L, normocytic anemia (hemoglobin of 10.1 g/dL), thrombocytosis (platelet count of 75 × 10^9^/L), and elevated CRP of 28 mg/L. CT demonstrated inflammation in the lungs and pleural effusion. We suspected infection by a drug-resistant microorganism and administered imipenem and methylprednisolone therapy. On day 9, her condition deteriorated and her respiratory rate increased with frequent cyanosis. The patient subsequently received tracheal intubation and mechanical ventilation. Because we could not exclude the possibility of methicillin-resistant *S. aureus* (MRSA) infection, vancomycin was administered. However, her oxygen saturation was only about 80%. X-ray showed interstitial emphysema, mediastinal emphysema, and subcutaneous emphysema in the lungs. She then received extracorporeal membrane oxygenation for 5 days. On day 22, bronchoscopy was performed, which revealed substantial purulent secretion and necrotic areas throughout the tracheal mucosa (Figure 2C1). Her condition improved and her tracheal tube was removed. Histologic evaluation showed inflammatory necrotic tissue and *Aspergillus fumigatus* infection (Figure 3C). Repeat CT showed local stenosis of right main bronchus (Figure 4C) and a cavity formed near the pleura in the right lower lung. She subsequently received voriconazole, caspofungin, and amphotericin B treatment. Bronchoscopy was performed seven times to remove the pseudomembrane. The last bronchoscopic examination during hospitalization showed tracheal stenosis and granulation (Figure 2C2). On day 44, she was discharged from our hospital and received oral voriconazole for 6 months. The last bronchoscopic examination showed persistence of some mucosal irregularities. CT taken 1 year after discharge showed almost complete absence of lung inflammation.

All patients were previously healthy and had no significant family history.

## Discussion

Pediatric pseudomembranous necrotizing tracheitis is rare. The disease was first reported as a complication of prolonged assisted ventilation in newborns in 1983 (3). Despite mucosal trauma from mechanical ventilation, mucosal damage from an antecedent viral infection may contribute to the pathogenesis of this disease (4, 5). The most common infectious etiologies reported were *Corynebacterium diphtheria, Corynebacterium pseudodiphtheriticum, S. aureus, Bacillus cereus, Pseudomonas aeruginosa, Aspergillus species, and Haemophilus influenza* (2, 5, 6). Opportunistic infection occurs in immunosuppressed patients such as those with hematologic malignancies, organ transplantation, or neutropenia (7). Pseudomembranous necrotizing tracheitis can also be a rare extra-intestinal manifestation of ulcerative colitis in children (8). This disease should be differentiated from acute laryngitis, epiglottitis, foreign body inhalation, and obstructive fibrinous tracheal pseudomembrane (9).

Bacterial bronchitis primarily affects preschool and early school-aged children, however, cases of children with necrotizing tracheitis are rarely reported (10). We searched the PubMed and Embase databases for articles published until December 30, 2019, using the following search terms: (necrotic OR necrotizing OR pseudomembranous OR aspergillus) AND (tracheitis OR tracheobronchitis). Neonatal necrotizing tracheitis was excluded. We only found seven cases of pediatric necrotizing tracheitis (Table 1). The exact incidence is unknown. All cases presented with high fever, cough, inspiratory stridor, hoarseness, and tachypnea. All patients had acute deterioration characterized by severe upper airway obstruction.

**Table 1:** Cases of children with necrotizing tracheitis.

**Number**	**Age**	**Sex**	**Etiology**	**Basic disease**	**Imaging**	**Endoscopic findings**	**Prognosis**	**Country**	**Reference**
1	3m	F	Unknown	Tetralogy of Fallot and absent pulmonary valve, a radical cure	Strong stenosis was supposed at the truncus intermedius	The mucosa was red with some scabs and the lumen was narrow with a lot of secretions	Died	Japan	(11)
2	8m	M	Unknown	No	Left lung was hyperinflation with mediastinal shifting	There were swelling, bleeding, necrosis, and scab in tracheal and bronchial mucosa	Well	Portugal	(12)
3	9y	F	Influenza A and methicillin-resistant *staphylococcus aureus*	No	Chest X-ray revealed there was bilateral patchy infiltration	Copious dark and cloudy secretions, ragged, and severely edematous with adherent fibrinous debris and patchy plaques	Died	USA	(13)
4	5y	F	Aspergillosis	Fanconi anemia	CT of the thorax revealed bibasilar pulmonary opacities	A white exophytic lesion was in the tracheal	Died	Colombia	(14)
5	9y	F	Aspergillosis	Chronic myelogenous leukemia, hematopoietic cell transplantation	Chest X-ray was normal. Lateral neck films showed subglottic airway narrowing with soft tissue fullness of the glottis and subglottic areas	There were an erythematous and edematous supraglottis and extensive pseudomembranous and obstructive tracheitis, an estimated 50–60% of the entire tracheal lumen was filled	Died	USA	(15)
6	16y	F	Unknown	Ulcerative colitis	Not mentioned	There were mucous ulceration and white plaques along tracheal	Well	Portugal	(8)
7	7y	F	Influenza A-H1N1 combined with *staphylococcus aureus*	No	Chest X-ray showed there was inflammation in the right lung	There were black and yellow necrotic substances in the main airway, accompanied by hemorrhage and a large amount of yellow purulent substances	Died	China	(16)

In the present report, all patients were girls aged 1–3 years old. The first two patients got ill in the spring, while the third fell ill in the summer. The infectious etiologies of the three cases were MSSA secondary to influenza A virus, and *Aspergillus* sp. However, the mechanism by which these infections progressed to necrotizing tracheitis was not entirely clear. Although community-acquired *S. aureus* strains isolated from the first two patients were sensitive to methicillin, the strains likely had stronger virulence than the typical MSSA strains. Case 2 had coinfection of influenza A virus and *S. aureus*. These organisms are known to have destructive synergism. For instance, *S. aureus* strain was shown to secret proteases capable of enhancing influenza infectivity and pathogenicity in the respiratory tract5. In case 3, Aspergillus infection may have resulted from the patinet's long-term use of antibiotics and hormones, and neutropenia. The course of disease for *Aspergillus* infection is longer and the prognosis is relatively poorer than other etiologies.

Imaging features of pseudomembranous necrotizing tracheitis include circumferential thickening of the trachea, tracheal stenosis, and diffuse haziness and irregularity of the tracheal wall (13). The CT scan of case 1 showed membranous material compatible with endoluminal densities that was strongly adherent to the tracheal wall below the vocal cord. All cases had radiological findings suggestive of pneumonia.

Microscope images can aid in the correct diagnosis of patients with complex respiratory conditions of similar presentation (1, 14). Bronchoscopic findings consisted of membranous plaques, thick respiratory secretions, ulceration, or denudation of airway mucosa (15). Endoscopy (flexible or rigid) also has an important role in both tracheal and bronchial tissue sampling for microscopic analysis and culture, and helps to avert the need for intubation (17). Treatment in the acute phase of the illness frequently requires insertion of an endotracheal tube into the inflamed airway, which may lead to the subsequent development of mucosal shedding and subglottic stenosis. Once a patient exhibited signs and symptoms of central airway obstruction, he or she often needs immediate bronchoscopic intervention to restore airway patency (2). Rigid bronchoscopy is the safest treatment modality, both for confirming and relieving airway obstruction in patients with acute respiratory failure (18, 19). It can maintain airway open, diagnose quickly and relieve airway obstruction in time. When the patient deteriorated or his vital signs were unstable, rigid bronchoscopy will be the first choice.

Given the concern about early scar formation and recurrent airway obstruction, it has been suggested that early flexible bronchoscopy should be performed in patients with evidence of severe tracheitis (2). In this report, repeated resection of pseudomembranous was often carried out under flexible bronchoscopy.

Case 1 was our first experience of necrotizing tracheitis. The patient only received endoscopic examination twice. When her situation worsened, her parents refused intervention by rigid endoscopy. She subsequently died of profound mucosal sloughing, which almost completely obstructed the main airway. The other two cases received endoscopic examination and mechanical debridement several times and showed a significantly improved prognosis. Therefore, awareness and early recognition and therapeutic intervention are essential to improved prognosis and prevent progression to respiratory failure in pediatric pseudomembrance necrotizing tracheitis.

Broad-spectrum intravenous antibiotic therapy should be initiated as soon as the clinical diagnosis is made. A third-generation cephalosporin agent combined with a beta-lactamase resistant penicillin is appropriate for first-line therapy (20). If MRSA is suspected, then vancomycin or linezolid should be chosen. Antibiotic coverage should also be modified according to culture. Voriconazole is currently the first choice for the treatment of invasive aspergillosis (7). Corticosteroids treatment remains controversial. However, based on the rationale that they reduce airway inflammation and oedema, it was used in most of patients (21).

## Conclusion

Pseudomembrance necrotizing tracheitis should be considered in children who present with an acute, life-threatening upper airway obstruction. Improvement in the mortality rate of this disease requires early recognition and prompt treatment with mechanical debridement by endoscopy and early initiation of broad-spectrum antibiotic therapy.

## Ethics Statement

Written informed consent was obtained from the minor(s)' legal guardian/next of kin for the publication of any potentially identifiable images or data included in this article.

## Author Contributions

ZC supervised research work. XW and LW participated in collecting information of three cases and searched for literatures. They contributed equally to this work. All authors participated in the interpretation of the data. All authors read and approved the final manuscript.

## Conflict of Interest

The authors declare that the research was conducted in the absence of any commercial or financial relationships that could be construed as a potential conflict of interest.
