# Supplementary material for: The Important Role of Endoscopy in Management of Pediatric Pseudomembranous Necrotizing Tracheitis
Source: Front Pediatr. 2020 Jul 9;8:360. doi: 10.3389/fped.2020.00360 (PMC7363969; doi:10.3389/fped.2020.00360)
Supplement: Supplementary file 1 [file Table_1.DOCX]

**CARE Checklist – 2016: Information for writing a case report**

**Topic Item Checklist item description Line/Page**

**Title 1** The words “case report” should be in the title along with the area of focus yes

**Key Words 2** Four to seven key words—include “case report” as one of the key words yes

**Abstract 3a** Background: What does this case report add to the medical literature? yes

**3b** Case summary: chief complaint, diagnoses, interventions, and outcomes yes

**3c** Conclusion: What is the main “take-away” lesson from this case? yes

**Introduction 4** The current standard of careand contributions of this case—with references (1-2 paragraphs) 29-39lines _

**Timeline 5** Information from this case report organized into a timeline (table or figure) described in days of _hospitation__

**Patient Information 6a** De-identified demographic and other patient or client specific information __Yes__

**6b** Chief complaint—what prompted this visit 42-43,75-76,100lines__

**6c** Relevant history including past interventions and outcomes 127line

**Physical Exam 7** Relevant physical examination findings 44,77,104lines

**Diagnostic 8a** Evaluations such as surveys, laboratory testing, imaging, etc. 46-50 lines

**Assessment 8b** Diagnostic reasoning including other diagnoses considered and challenges 59-62lines

**8c** Consider tables or figures linking assessment, diagnosesand interventions No

**8d** Prognostic characteristics where applicable 71,97,125lines

**Interventions 9a** Types such as life-style recommendations, treatments, medications, surgery 48-68,83-94,120lines

**9b** Intervention administration such as dosage, frequency and duration _90,120lines_

**9c** Note changes in intervention with explanation _Yes_____

**9d** Other concurrent interventions __Yes_____

**Follow-up and 10a** Clinician assessment (and patient or client assessed outcomes when appropriate) 71,97,125lines

**Outcomes 10b** Important follow-up diagnostic evaluations 71,97,125lines

**10c** Assessment of intervention adherence and tolerability, including adverse events _No______

**Discussion 11a** Strengths and limitations in your approach to this case 182-189lines

**11b** Specifyhow this case report informs practice or Clinical Practice Guidelines (CPG) _189______

**11c** How does this case report suggest a testable hypothesis? 187-189lines

**11d** Conclusions and rationale 198-201 lines

**Patient Perspective 12** When appropriate include the assessment of the patient or client on this episode of care ____No___

**Informed Consent 13** Informed consent from the person who is the subject of this case report is required by most journals ___Yes____

**Additional Information 14** Acknowledgement section; Competing Interests; IRB approval when required ____Yes___
